# MySurgeryRisk Model Predictions of Postoperative Complications and Mortality

**DOI:** 10.1001/jamasurg.2026.1112

**Published:** 2026-04-29

**Authors:** Yuanfang Ren, Esra Adiyeke, Ziyuan Guan, Zhenhong Hu, Tyler J. Loftus, Benjamin Shickel, Parisa Rashidi, Tezcan Ozrazgat-Baslanti, Azra Bihorac

**Affiliations:** 1 Intelligent Clinical Care Center, University of Florida, Gainesville, Florida; 2 Division of Nephrology, Hypertension, and Renal Transplantation, Department of Medicine, University of Florida, Gainesville, Florida; 3 Department of Surgery, University of Florida, Gainesville, Florida; 4 Department of Biomedical Engineering, University of Florida, Gainesville, Florida

## Abstract

**Importance:**

Despite advances in surgical techniques and care, postoperative complications are prevalent and affect up to 15% of patients who undergo inpatient surgery. Analytic models for surgical risk estimation can benefit from incorporating complex electronic health record data across multiple, diverse settings.

**Objective:**

To test the hypothesis that applying the MySurgeryRisk framework, developed and prospectively validated on a single-center dataset, to a large multicenter dataset will enhance generalizability without degrading the predictive performance achieved by the original, single-center model.

**Design, Setting, and Participants:**

This retrospective, longitudinal, multicenter cohort analysis included 508 097 encounters from 366 875 adult patients admitted and underwent major inpatient operations at 14 health care institutions within the OneFlorida + network from 2012 through 2023. Models were trained on a development set (2012-2020; n = 358 216 encounters) and evaluated on a validation set (2020-2023; n = 149 881 encounters). These data were analyzed from January 2025 to February 2026.

**Main Outcomes and Measures:**

Using the feature selection and transformation methods validated in the MySurgeryRisk framework, eXtreme Gradient Boosting models were developed and validated to predict the postoperative risk of intensive care unit (ICU) admission, postoperative mechanical ventilation (MV), postoperative acute kidney injury (AKI), and in-hospital mortality. Performance was primarily evaluated using area under the receiver operating characteristics curve (AUROC) values.

**Results:**

Among 366 875 total patients who underwent 508 097 major inpatient operations, the mean (SD) age was 59 (18) years; 190 799 (52%) were women and 176 076 were men (48%). The prevalence of complications was 8% (n = 42 302) for ICU admission, 4% (n = 20 435) for MV, 7% (n = 36 027) for AKI, and 1% (n = 5131) for in-hospital mortality. AUROC values were as follows: ICU admission, 0.93 (95% CI, 0.93-0.93); MV, 0.94 (95% CI, 0.94-0.94); AKI, 0.92 (95% CI, 0.92-0.92); and in-hospital mortality, 0.95 (95% CI, 0.94-0.95). Model predictive performance was comparable with previously validated MySurgeryRisk models. Primary procedure code and clinician-specific factors were consistently the most influential variables.

**Conclusions and Relevance:**

A model using routinely collected variables from a large multicenter cohort was developed and validated, demonstrating accurate prediction of postoperative complications and death with generalizability across a large network of health care institutions. Procedure type and clinician-specific factors were the most influential contributors to predicted risk, providing insights into factors influencing surgical outcomes.

## Key Points

**Question:**

Will applying the MySurgeryRisk framework, originally developed and prospectively validated on a single-center dataset, to a large multicenter dataset enhance generalizability without degrading performance?

**Findings:**

The models predicted postoperative complications in this cohort analysis of 508 097 encounters from 366 875 patients who underwent major operations at 14 health care institutions. Model performance was comparable with the previously validated MySurgeryRisk models, with procedure type and clinician-specific factors identified as the most influential contributors to predicted risk.

**Meaning:**

In this study, incorporating routinely collected variables from a multicenter cohort enabled highly generalizable, accurate prediction of postoperative complications.

## Teaser

This study tests the hypothesis that applying the MySurgeryRisk framework to a large multicenter dataset will enhance generalizability without degrading the predictive performance achieved by the original, single-center model.

## Introduction

Over 310 million major operations are performed globally each year.^1^ Despite advances in surgical techniques and perioperative care, postoperative complications still occur in up to 15% of cases and overall postoperative mortality ranges from 0.79% to 5.7%.^2^ These complications adversely affect patients’ quality of life and are associated with increased risk of reoperation, extended hospital stay, and higher mortality rates.^3,4,5^ Notably, postoperative complications impact survival after major surgery more than preoperative risk factors or intraoperative events. Furthermore, postoperative complications impose a major financial burden, primarily due to intensive care unit (ICU) admission or rehospitalization.^2,6,7^

Key determinants of postoperative complication risk include patients’ age, comorbidities, and care provision.^5,8^ Accurate risk estimation is essential for identifying patients who would benefit from risk-mitigation strategies; however, traditional methods struggle to analyze the vast, complex data within electronic health records (EHRs). While digital tools leveraging EHRs are increasingly vital, current models^9,10,11^ often depend on largely inaccessible information in EHRs or have not been adapted for full integration into clinical workflows.

To address these challenges, we previously developed MySurgeryRisk, a machine-learning platform that demonstrated excellent performance by automating the analysis of large-scale EHR data.^12,13,14^ However, its development on a single-institution cohort potentially limits its generalizability. We hypothesize that applying the MySurgeryRisk framework to a large, multicenter dataset will yield models with enhanced generalizability without degrading performance. To test this, we used data from the OneFlorida Data Trust—a clinical data research network comprising 14 health systems—to develop and validate MySurgeryRisk model for predicting risk of postoperative ICU admission, mechanical ventilation (MV), acute kidney injury (AKI), and in-hospital mortality.

## Methods

### Study Design and Participants

**Figure 1.**
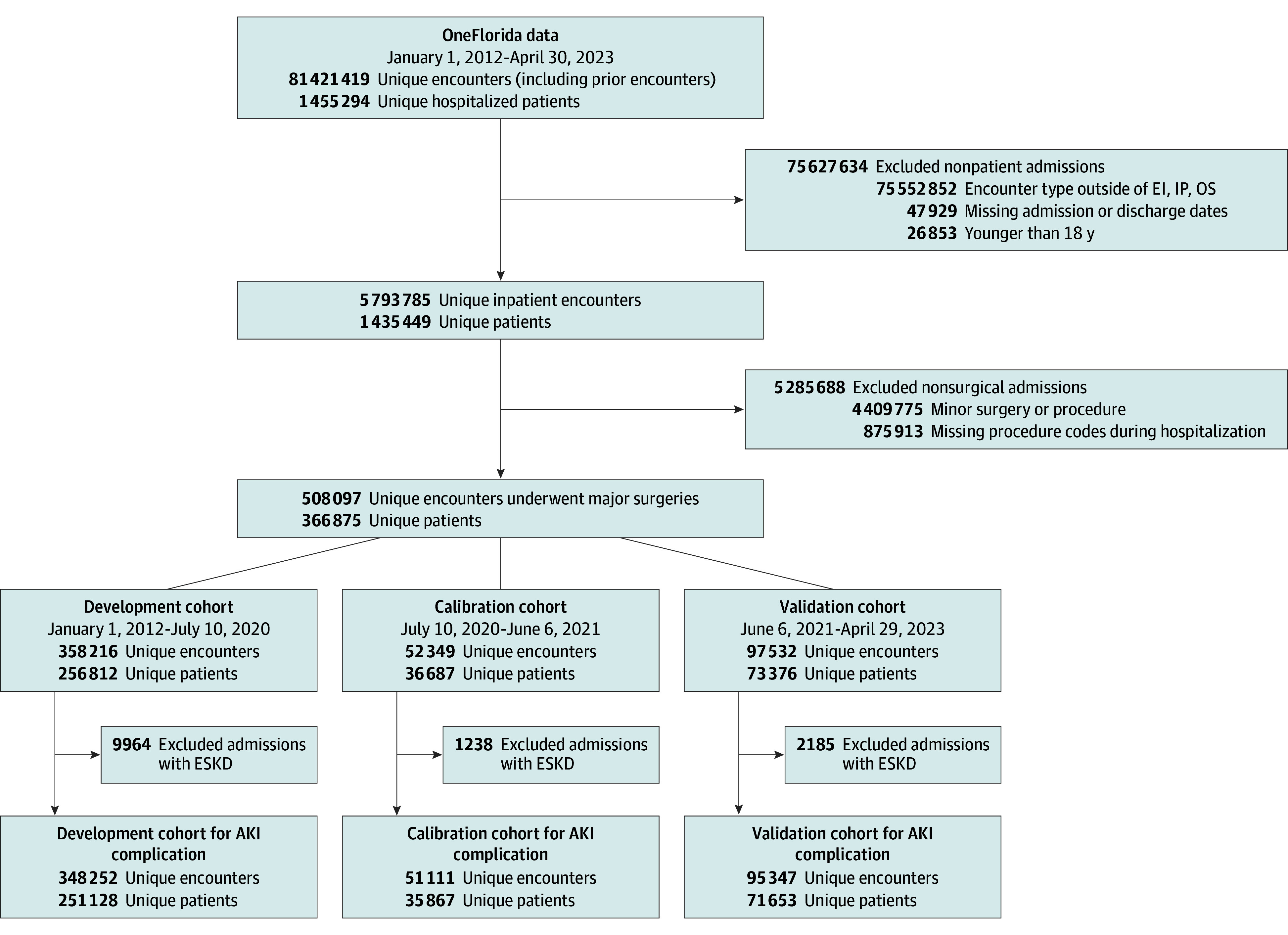
Flow Diagram of MySurgeryRisk Model Development and Validation Cohorts AKI indicates acute kidney injury; EI, emergency department admit to inpatient hospital stay; ESKD, end-stage kidney disease; IP, inpatient hospital stay; OS, observation stay.

We obtained longitudinal EHR data for 1 455 294 hospitalized patients admitted to health care institutions within the OneFlorida + network between January 1, 2012, and April 30, 2023, comprising 81 421 419 admissions (including historical admissions). We excluded outpatient admissions, patients younger than 18 years, and those who did not undergo major surgery (Figure 1). Our final cohort included 508 097 encounters from 366 875 patients. We excluded patients with end-stage kidney disease when predicting AKI complication. The study was approved by the University of Florida Institutional Review Board and Privacy Office (202300641) as an exempt study with a waiver of informed consent.

Following guidelines in Transparent Reporting of a Multivariable Prediction Model for Individual Prognosis or Diagnosis (TRIPOD) reporting guidelines^15^ under the Type 2b analysis category, we chronologically split the cohort into development (70% of observations; n = 358 216 encounters), calibration (10%; n = 52 349), and validation (20%; n = 97 532) sets. We used the development cohort for model development and parameter selection, the calibration cohort for model calibration, and the validation cohort for model performance assessment.

### Identification of Major Surgery and Outcomes

We identified major surgeries using *Current Procedural Terminology* (*CPT*) codes and associated relative value units (RVUs). We selected *CPT* codes where the associated RVUs included an intraoperative portion and were classified as major surgery (eFigure 1 in Supplement 1). When a patient had multiple surgeries during 1 admission, only the surgery with maximum intraoperative working units was included in the analysis. The primary outcomes were postoperative ICU admission, MV, AKI, and in-hospital mortality (detailed in the eMethods and eTable 1 in Supplement 1).

### Predictors

The previous MySurgeryRisk model^14^ used 137 input features, including preoperative demographic, socioeconomic, administrative, clinical, pharmacy, and laboratory variables. From these, we selected 99 input features, excluding those not available in the OneFlorida dataset, including detailed surgery information (eg, anesthesia type), those not routinely collected (eg, smoking status), variables limiting generalizability (eg, surgeon identity, zip code), and redundant laboratory metrics. eTable 2 in Supplement 1 lists the input features and their missingness.

Data preprocessing followed previously described methods, including outlier removal (top and bottom 1%) and expert-guided data cleaning.^12,14^ Missing categorical variables were treated as a distinct missing category, while missing continuous variables were imputed using the median of the development cohort. For high-dimensional categorical variables (ie, primary procedure code), we transformed these variables into conditional probabilities using the log of conditional odds of positive outcome.^14^

### Statistical Methods

We developed eXtreme Gradient Boosting (XGBoost) models for predicting postoperative complications. We calibrated the model using the calibration cohort with Platt scaling^16^ and isotonic regression^17^ methods. We selected the best-calibrated model based on Brier score^18^ and reliability plots.^19^ We identified important features contributing to predictions using SHAP (Shapley Additive Explanations).^20^ We further conducted an error subgroup analysis using a decision tree regressor to identify clinical blind spots where model reliability diminished (eMethods in Supplement 1). We assessed the robustness of models through subgroup analyses (by sex, race, and age) and a sensitivity analysis incorporating surgeon identity as a personalized feature. We evaluated model performance using area under the receiver operating characteristics curve (AUROC), area under the precision-recall curve (AUPRC), sensitivity, specificity, positive predictive value (PPV), negative predictive value (NPV), the Brier score, and reliability plots. Refer to the eMethods in Supplement 1 for further detail.

## Results

### Patient Baseline Characteristics and Outcomes

In the development cohort (256 812 total patients with 358 216 major surgical procedures), the mean (SD) age was 58 (19) years; 121 523 patients (47%) were male and 135 289 (53%) were female; 50 910 patients (20%) self-reported race as African American, 187 883 (73%) as White, 10 105 as other race (4%), and race data were missing for 7914 patients (3%) (Table 1). The validation cohort (73 376 patients with 97 532 major surgical procedures) exhibited statistically significant differences in demographic characteristics compared with the development cohort. This cohort had a mean (SD) age of 58 (17) years and a slightly lower proportion of female patients (50%). A slightly lower proportion of patients were African American (17%), and a slightly higher proportion of patients were White (75%).

**Table 1.** Summary of Baseline Characteristics and Outcomes

Variables	No. (%)			*P* value
	Development cohort	Calibration cohort	Validation cohort	
No. of patients	256 812	36 687	73 376	
No. of surgical procedures	358 216	52 349	97 532	
Age, mean (SD), y^a^	58 (19)	58 (17)^f^	58 (17)^f^	<.001
Sex^a^				
Male	121 523 (47)	18 073 (49)^f^	36 480 (50)^f^	<.001
Female	135 289 (53)	18 614 (51)^f^	36 896 (50)^f^	<.001
Race^a^^,^^b^				
African American	50 910 (20)	6645 (18)^f^	12 837 (17)^f^	<.001
White	187 883 (73)	27 587 (75)^f^	55 328 (75)^f^	<.001
Other^c^	10 105 (4)	1378 (4)	3148 (4)^f^	<.001
Missing	7914 (3)	1077 (3)	2063 (3)^f^	<.001
Ethnicity^a^^,^^b^				
Hispanic	45 074 (18)	9245 (25)^f^	19 467 (27)^f^	<.001
Non-Hispanic	203 676 (79)	25 731 (70)^f^	50 586 (69)^f^	<.001
Missing	8062 (3)	1711 (5)^f^	3323 (5)^f^	<.001
Insurance^a^				
Private	111 464 (43)	18 383 (50)^f^	36 664 (50)^f^	<.001
Medicare	84 681 (33)	10 173 (28)^f^	19 977 (27)^f^	<.001
Medicaid	29 208 (11)	3275 (9)^f^	6059 (8)^f^	<.001
Uninsured	31 459 (12)	4856 (13)^f^	10 676 (15)^f^	<.001
Complications^d^				
Postoperative intensive care unit admission	27 656 (8)	5036 (10)^f^	9610 (10)^f^	<.001
Postoperative mechanical ventilation	12 577 (4)	2980 (6)^f^	4878 (5)^f^	<.001
Postoperative acute kidney injury^e^	25 719 (7)	3414 (7)^f^	6894 (7)	<.001
In-hospital mortality	3865 (1)	469 (1)^f^	797 (1)^f^	<.001

^a^ Data were reported based on values calculated at the latest hospital admission.

^b^ Race and ethnicity were self-reported.

^c^ Other races include American Indian or Alaska Native, Asian, Native Hawaiian or Pacific Islander, and multiracial.

^d^ Data were reported based on postoperative complication status for each surgical procedure. When a patient had multiple surgeries during 1 admission, only the surgery with maximum intraoperative working units was used in the analysis.

^e^ The percentage was calculated after excluding patients with end-stage kidney disease. The numbers of surgical procedures used for postoperative acute kidney injury prediction in development, calibration, and validation cohorts were 348 252, 51 111, and 95 347, respectively.

^f^ The *P* values represent difference <.05 compared with the development cohort and were adjusted for multiple comparisons using Bonferroni method.

The prevalence of postoperative complications in the development cohort was 8% for postoperative ICU admission, 4% for MV, 7% for AKI, and 1% for in-hospital mortality. There was slight variation in complication prevalence between the validation and development cohorts (eg, prevalence was 10% for ICU admission and 5% for MV in the validation cohort).

### Model Performance

We selected the isotonic regression method for model calibration because the calibrated model demonstrated a lower Brier score (eTable 3 in Supplement 1) and close alignment in the reliability plot (eFigure 2 in Supplement 1) for the calibration cohort. We reported calibrated models’ performance in predicting postoperative complications on the hold-out validation cohort and presented the results for AUROC, AUPRC, sensitivity, specificity, PPV, and NPV with 95% CIs in Table 2, along with the calibration performance (Table 2; eFigure 3 in Supplement 1). AUROC values ranged from 0.92 to 0.95: ICU admission, 0.93 (95% CI, 0.93-0.93); MV, 0.94 (95% CI, 0.93-0.94); AKI, 0.92 (95% CI, 0.91-0.92); and in-hospital mortality, 0.95 (95% CI, 0.94-0.95). AUPRC values ranged from 0.25 to 0.61: ICU admission, 0.61 (95% CI, 0.60-0.63); MV, 0.44 (95% CI, 0.42-0.45); AKI, 0.54 (95% CI, 0.52-0.55); and in-hospital mortality, 0.25 (95% CI, 0.22-0.29). Reliability plots revealed high fidelity between predicted and observed probabilities, particularly in the low- to moderate risk ranges (0%-40%) for all outcomes. While ICU admission and AKI models maintained near-perfect calibration across the entire risk spectrum, the MV therapy and in-hospital mortality models exhibited a trend toward over prediction at the highest risk deciles (>50%).

**Table 2.** Model Performance Measurements for Postoperative Complications With 95% CI in the Validation Cohort

Complications	(95% CI)							Yoden Index
	AUROC	AUPRC	Sensitivity	Specificity	PPV	NPV	Brier score	
ICU admission	0.93 (0.93-0.93)	0.61 (0.60-0.63)	0.87 (0.85-0.89)	0.82 (0.81-0.84)	0.35 (0.34-0.37)	0.98 (0.98-0.99)	0.06 (0.05-0.06)	0.09
MV	0.94 (0.93-0.94)	0.44 (0.42-0.45)	0.88 (0.86-0.89)	0.86 (0.86-0.88)	0.25 (0.24-0.27)	0.99 (0.99-0.99)	0.04 (0.04-0.04)	0.04
AKI	0.92 (0.91-0.92)	0.54 (0.52-0.55)	0.86 (0.85-0.88)	0.8 (0.78-0.82)	0.25 (0.24-0.27)	0.99 (0.99-0.99)	0.05 (0.05-0.05)	0.1
In-hospital mortality	0.95 (0.94-0.95)	0.25 (0.22-0.29)	0.91 (0.85-0.93)	0.84 (0.84-0.91)	0.04 (0.04-0.07)	1.0 (1.0-1.0)	0.01 (0.01-0.01)	0.01

Abbreviations: AKI, acute kidney injury; AUROC, area under the receiver operating characteristic curve; AUPRC, area under the precision-recall curve; ICU, intensive care unit; MV, mechanical ventilation; NPV, negative predictive value; PPV, positive predictive value.

**Figure 2.**
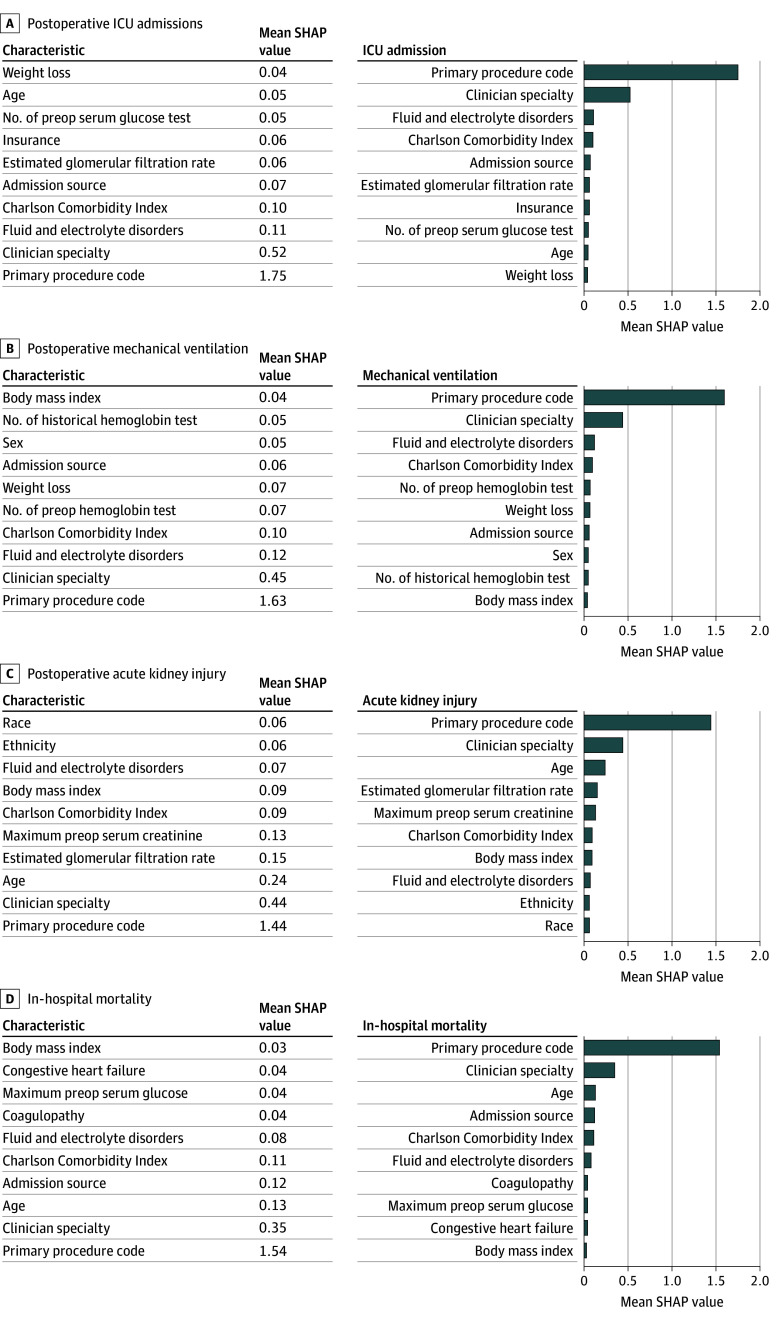
Bar Graphs Showing Top 10 Important Features Contributing to the Prediction for Postoperative Complications The x-axis represents the mean absolute SHAP (Shapley Additive Explanations) values, which quantify the average contribution of each feature to the prediction model. Higher mean SHAP values indicate that a feature has a greater impact on the model’s predictions for postoperative complications. ICU indicates intensive care unit.

Figure 2 presents important features contributing to the model predictions for postoperative complications. Across all complications, primary procedure code was consistently the most important feature, indicating that the type of surgical procedure is the strongest predictor for postoperative complications. Clinician specialty also consistently appeared as the second most important feature, suggesting that the clinician’s expertise and specialization play a critical role. Comorbidity-related features, including fluid and electrolyte disorders and Charlson Comorbidity Index, were ranked as important predictors across complications. The feature admission source (whether the patient was transferred from another facility) may indicate an urgent admission and was an important predictor for all complications, except for AKI. Age, estimated glomerular filtration rate, and maximum preoperative serum creatinine were important for predicting AKI, highlighting the relevance of kidney function metrics. For in-hospital mortality, comorbidities like coagulopathy and congestive heart failure played significant roles.

eFigures 4-7 in Supplement 1 present the error analysis results across outcomes. The models exhibited consistent blind spots among patients transferred from external facilities, those undergoing low-acuity procedures with intensive monitoring, and those with significant baseline metabolic or physiological derangements.

### Robustness of Models

eTables 4-6 in Supplement 1 present model discrimination with respect to patient demographic profiles (sex, race, and age). Across all models, results consistently showed similar or better AUROC, sensitivity, specificity, NPV, and Brier score for female patients compared with male patients, but similar or worse AUPRC and PPV. Similarly, when comparing African American patients and non–African American patients, the model consistently demonstrated similar or better AUROC, sensitivity, specificity, NPV, and Brier score, but similar or lower AUPRC and PPV for non–African American patients across all complications. Model performance across age groups varied depending on the complication. For younger patients compared with older patients, the model exhibited distinct trends: it showed higher AUPRC, specificity, and PPV but lower sensitivity for ICU admission; higher AUPRC for MV; higher specificity but lower AUPRC and PPV for AKI; and higher AUROC, AUPRC, and sensitivity but lower PPV for in-hospital mortality.

**Figure 3.**
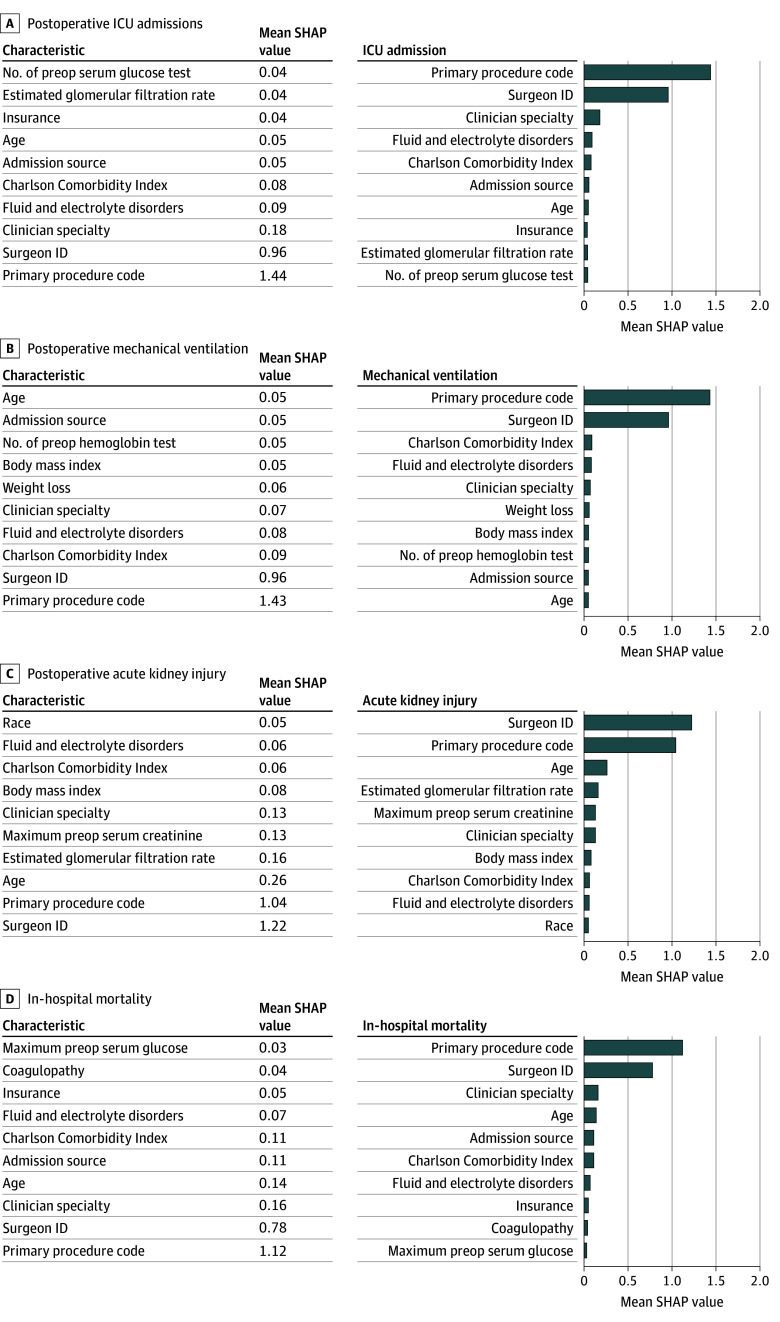
Bar Graphs Showing Sensitivity Analysis Adding Personalized Feature Surgeon Identity: Top 10 Important Features Contributing to the Prediction for Postoperative Complications The x-axis represents the mean absolute SHAP (Shapley Additive Explanations) values, which quantify the average contribution of each feature to the prediction model. Higher mean SHAP values indicate that a feature has a greater impact on the model’s predictions for postoperative complications. ICU indicates intensive care unit; ID, identity.

A sensitivity analysis was conducted by incorporating a personalized feature (surgeon identity) in model development to determine its impact (eTable 7 in Supplement 1). Across all complications, the performance of the models with and without surgeon identity remained nearly consistent, with slight difference in sensitivity and specificity. The model including surgeon identity achieved slightly higher specificity for ICU admission, higher sensitivity but lower specificity for AKI, and higher specificity but lower sensitivity for in-hospital mortality. Figure 3 presents the important features for the model including surgeon identity. Surgeon identity replaced clinician specialty as the second most important feature for almost all complications and became the top predictor for AKI. In the model without surgeon identity, the primary procedure code dominated predictive contribution; with surgeon identity, the surgeon’s identifier showed higher importance. The remaining important features and their ranking remained largely unchanged.

## Discussion

In this study, we applied our MySurgeryRisk framework to a large multicenter dataset to develop prediction models for postoperative complications. The developed models achieved high AUROC values. Feature importance analysis demonstrated that type of surgery and clinician-specific factors played significant roles in predicting postoperative complication risk.

MySurgeryRisk algorithm is an automated EHR-based tool integrated into an intelligent perioperative platform, delivering real-time risk predictions via web and mobile interfaces. Compared with the widely used the American College of Surgeons (ACS) National Surgical Quality Improvement Program (NSQIP) calculator, which requires manual entry of limited variables, MySurgeryRisk leverages the full depth of the EHR to automate risk assessment without increasing clinician burden. Despite its vision, clinicians in our codesign sessions identified the ACS NSQIP calculator as the most commonly used tool due to its perceived credibility and extensive multicenter validation.^21^ Conversely, previous versions of MySurgeryRisk faced adoption barriers, where one primary concern is that the model population was from a single institution limiting its generalizability.^21^ This feedback prompted the current iteration; by leveraging the OneFlorida Data Trust, we have transitioned the algorithm to a statewide, multicenter framework. We refined the model to rely exclusively on routinely collected EHR features, ensuring seamless automated deployment without local data-entry barriers. Architecturally, we transitioned the model’s architecture from Random Forest to XGBoost to better handle the high-dimensional, sparse EHR data inherent in multicenter datasets. We also refined the outcomes to acute, in-hospital metrics. Despite these adjustments, our developed models demonstrated excellent performance with AUROCs from 0.92 through 0.95, comparable with the single-center version across similar complications (AUROCs of 0.82-0.91). Reliability plots show high fidelity, with MV/in-hospital mortality over prediction at high-risk potentially, signaling effective clinical vigilance. Beyond statistical refinement, this iteration meaningfully improves clinical relevance by addressing the generalizability gap—providing the robust, external validation necessary for surgeons to trust the tool across heterogeneous clinical environments.

Feature importance analysis revealed that type of surgery, clinician specialty, and patient’s health condition play significant roles in predicting postoperative complication risk, aligning with previous studies.^5,14,22^ Dharap et al^22^ demonstrated that comorbidities, higher surgical risk per American Society of Anesthesiologists grading, higher body mass index, surgery type, and other intraoperative surgical features were significant risk factors for postoperative complications. Expanding on this, our model comparison showed that the inclusion of a more granular clinician-specific predictor, surgeon ID, which reflects a surgeon’s historical performance in relation to their case mix, demonstrated robust performance in AUROC and AUPRC, and slight performance improvement in sensitivity and specificity for most complications. Furthermore, when included, surgeon ID emerged as a primary predictor, often surpassing traditional clinical comorbidities. This finding underscores the significant impact of clinician-specific factors, such as technical proficiency, surgical volume, and perioperative management, on patient recovery. While patient comorbidities are frequently nonmodifiable, the identification of surgeon-level variance provides a critical pathway for systemic quality improvement. These data enable hospitals to implement risk-stratified triage, ensuring high-risk patients are matched with surgical teams whose expertise aligns with the complexity of the case. Ultimately, by accounting for these clinician effects, the MySurgeryRisk model offers a more transparent baseline for informed consent, allowing for a realistic reconciliation between a patient’s physiological risk and the anticipated surgical performance.

The identification of clinical blind spots suggests that while the models are highly reliable for the average surgical patient, their utility may be limited in cases of extreme physiological instability or data fragmentation. For instance, the underperformance in transferred patients likely stems from *information decay*, where critical pretransfer clinical nuances are lost in EHR transmission. These findings underscore that artificial intelligence–based risk tools should supplement, rather than replace, clinical intuition in high-complexity or data-poor scenarios. Subgroup analysis across patient demographic profiles revealed the models exhibited slightly better performance for female patients, non-African American patients, and younger patients. Additionally, we observed significant differences in demographic characteristics and outcome prevalence between the development and validation cohorts—specifically an increase in ICU admission and MV—which are likely attributed to data drift caused by the COVID-19 pandemic. During this crisis state, hospital programs prioritized high-acuity, medically necessary procedures, resulting in a validation cohort with a higher baseline risk and different insurance profiles than the prepandemic development set.

Our MySurgeryRisk platform is designed to function as a dynamic clinical decision support tool across the entire preoperative continuum. Its primary utility lies in preoperative triage and procedure risk communication, where real-time, personalized risk scores allow surgeons and patients to engage in more transparent shared decision-making. For patients identified as high risk—determined by the optimized Youden Index threshold in our model—the algorithm serves as an automated trigger for operative optimization consults, such as geriatric comanagement, nutritional support, or specialized prehabilitation programs. At the bedside, these data-driven insights enhance procedural risk communication by providing a standardized baseline that reconciles a patient’s physiological complexity with the anticipated surgical performance. Future inclusion of uncertainty quantification will further assist these discussions by signaling model confidence. Furthermore, by predicting the immediate requirement for ICU admission or MV, the tool also allows for better perioperative planning and resource allocation for high-acuity patients. Clinicians can allocate ICU beds, adjust staff requirements, and ensure the availability of specialized care based on predictive needs. To translate MySurgeryRisk from a validated predictive model to a clinical intervention, we have defined a structured implementation road map that builds on the foundational work established with previous versions.^12,14,23^ Prior work with the single-center version of MySurgeryRisk established the technical feasibility and clinical acceptance of the tool. A pilot study demonstrated that surgeons’ risk-assessment accuracy significantly improved (with net reclassification improvements of 12% for AKI and 16% for ICU admissions) after interacting with the algorithm.^23^ A prospective validation of MySurgeryRisk demonstrated that automated real-time predictions of postoperative complications with mobile device outputs had good performance in clinical settings, matching surgeons’ predictive accuracy.^14^ The release of this multicenter iteration enables the measurement of workflow integration metrics and clinician adoption metrics across diverse settings. Initial pilot deployment will focus on measuring the frequency of risk report engagement and the impact of the mobile interface on preoperative consult efficiency. In the next phase, to formally evaluate the model’s impact on patient outcomes, we propose a stepped-wedge cluster randomized trial across OneFlorida Data Trust institutions. This design allows for a robust comparison of complication rates, length of stay, and resource utilization as the tool is sequentially activated at each site. To ensure version stability during this formal evaluation, the prediction logic will remain fixed within 12-month cycles. However, recognizing the potential for data drift, the algorithm will undergo annual retraining using the latest data.

### Limitations

Our study has several limitations. First, the dataset lacks detailed information about the surgeries. As a result, identifying major surgeries and determining their exact start and end times based solely on *CPT* codes can introduce bias into the dataset. Additionally, the estimated surgery start and end times, coupled with the absence of other data (ie, station and respiratory device information) limit the scope of our outcomes. Consequently, the 8 postoperative complications and mortality metrics in previous studies have been reduced and prolonged ICU stay and MV (greater than 48 hours) have been redefined as mere ICU admission and MV requirements. These limitations affect the generalizability of our models. Future studies should focus on creating a multicenter surgical dataset with clear provenance, using common data models, and containing abundant elements specific to surgeries, and diverse, multi-institutional data. Second, the demographic and clinical disparity between development and validation cohort indicates a significant data shift, which may have been influenced by the COVID pandemic. This data drift underscores the necessity for continuous model monitoring and periodic retraining to maintain predictive stability. Third, the models lack external validation, limiting generalizability. Last, the models still exhibited performance blind spots in specific high-complexity or data-limited subgroups (eg, interfacility transfers). Future iterations should focus on enhancing model fairness by incorporating more granular social determinants of health and refining features for these vulnerable clusters to prevent potential inequalities in surgical care.

## Conclusions

By applying the MySurgeryRisk framework to a large, multicenter dataset from OneFlorida Data Trust, we developed models using routinely collected clinical information to predict postoperative complications and death after major surgery with high performance and generalizability. Procedure type and clinician-specific factors were the most influential contributors to predicted risk, providing insights into factors influencing surgical outcomes. Further work is necessary to generate a comprehensive multicenter surgical dataset incorporating more granular, surgery-specific elements and to improve data and model fairness.

## Notes

Supplement 1. eMethods eFigure 1. Flow Diagram for the identification of major surgery and the surgical cohort eFigure 2. Reliability plot demonstrating calibration performance on the calibration cohort eFigure 3. Reliability plot demonstrating calibration performance on the validation cohort eFigure 4. Error subgroup analysis identifying clinical blind spots for intensive care unit (ICU) admission eFigure 5. Error subgroup analysis identifying clinical blind spots for postoperative mechanical ventilation (MV) eFigure 6. Error subgroup analysis identifying clinical blind spots for postoperative acute kidney injury (AKI) eFigure 7. Error subgroup analysis identifying clinical blind spots for in-hospital mortality eTable 1. Administrative codes used for intensive care unit admission and mechanical ventilation eTable 2. Input features used in models eTable 3. Brier score assessment of calibration methods in the calibration cohort eTable 4. Model performance measurements for postoperative complications with 95% confidence interval in the validation cohort stratified by sex eTable 5. Model performance measurements for postoperative complications with 95% confidence interval in the validation cohort stratified by race eTable 6. Model performance measurements for postoperative complications with 95% confidence interval in the validation cohort stratified by age eTable 7. Sensitivity analysis adding personalized feature surgeon ID: model performance measurements for postoperative complications with 95% confidence interval in the validation cohort

Supplement 2. Data sharing statement
