# Supplementary material for: MySurgeryRisk Model Predictions of Postoperative Complications and Mortality
Source: JAMA Surg. 2026 Apr 29;161(6):619–27. doi: 10.1001/jamasurg.2026.1112 (PMC13130070; doi:10.1001/jamasurg.2026.1112)
Supplement: Supplement 1. — eMethods eFigure 1. Flow Diagram for the identification of major surgery and the surgical cohort eFigure 2. Reliability plot demonstrating calibration performance on the calibration cohort eFigure 3. Reliability plot demonstrating calibration performance on the validation cohort eFigure 4. Error subgroup analysis identifying clinical blind spots for intensive care unit (ICU) admission eFigure 5. Error subgroup analysis identifying clinical blind spots for postoperative mechanical ventilation (MV) eFigure 6. Error subgroup analysis identifying clinical blind spots for postoperative acute kidney injury (AKI) eFigure 7. Error subgroup analysis identifying clinical blind spots for in-hospital mortality eTable 1. Administrative codes used for intensive care unit admission and mechanical ventilation eTable 2. Input features used in models eTable 3. Brier score assessment of calibration methods in the calibration cohort eTable 4. Model performance measurements for postoperative complications with 95% confidence interval in the validation cohort stratified by sex eTable 5. Model performance measurements for postoperative complications with 95% confidence interval in the validation cohort stratified by race eTable 6. Model performance measurements for postoperative complications with 95% confidence interval in the validation cohort stratified by age eTable 7. Sensitivity analysis adding personalized feature surgeon ID: model performance measurements for postoperative complications with 95% confidence interval in the validation cohort [file jamasurg-e261112-s001.pdf]

Supplemental Online Content

Ren Y, Adiyeke E, Guan Z, et al. MySurgeryRisk model predictions of postoperative complications and mortality. *JAMA Surg*. Published online April 29, 2026. doi:10.1001/jamasurg.2026.1112

eMethods
eFigure 1. Flow Diagram for the identification of major surgery and the surgical cohort
eFigure 2. Reliability plot demonstrating calibration performance on the calibration cohort
eFigure 3. Reliability plot demonstrating calibration performance on the validation cohort
eFigure 4. Error subgroup analysis identifying clinical blind spots for intensive care unit (ICU) admission
eFigure 5. Error subgroup analysis identifying clinical blind spots for postoperative mechanical ventilation (MV)
eFigure 6. Error subgroup analysis identifying clinical blind spots for postoperative acute kidney injury (AKI)
eFigure 7. Error subgroup analysis identifying clinical blind spots for in-hospital mortality
eTable 1. Administrative codes used for intensive care unit admission and mechanical ventilation
eTable 2. Input features used in models
eTable 3. Brier score assessment of calibration methods in the calibration cohort
eTable 4. Model performance measurements for postoperative complications with 95% confidence interval in the validation cohort stratified by sex
eTable 5. Model performance measurements for postoperative complications with 95% confidence interval in the validation cohort stratified by race
eTable 6. Model performance measurements for postoperative complications with 95% confidence interval in the validation cohort stratified by age
eTable 7. Sensitivity analysis adding personalized feature surgeon ID: model performance measurements for postoperative complications with 95% confidence interval in the validation cohort

This supplemental material has been provided by the authors to give readers additional information about their work.

© 2026 American Medical Association. All rights reserved, including those for text and data mining, AI training, and similar technologies.

© 2026 American Medical Association. All rights reserved, including those for text and data mining, AI training, and similar technologies.

eMethods

Identification of Major Surgery and Outcomes

We identified major surgeries using Current Procedural Terminology (CPT) codes and associated relative value units (RVUs). RVUs are a measure used in the United States healthcare system to quantify the value of medical services. Using RVUs as a guide, we selected CPT codes where the associated RVUs included an intraoperative portion and were classified as major surgery (eFigure 1). When a patient had multiple surgeries during one admission, only the surgery with maximum intraoperative working units was included in the analysis. Since exact dates and times for the start and end of surgeries were not available, we used the date associated with the CPT code to mark start and end dates of the surgery.

The primary outcomes were three postoperative complications: postoperative intensive care unit (ICU) admission, postoperative mechanical ventilation (MV), acute kidney injury (AKI) and in-hospital mortality. To ensure consistency across the 14 participating institutions, we leveraged the OneFlorida Data Trust, which utilizes the PCORnet Common Data Model (CDM). This framework standardizes heterogeneous EHR data into uniform diagnostic and laboratory-based criteria, significantly reducing the impact of local coding variations. Postoperative ICU admissions and MV were identified using diagnosis and procedure codes (eTable 1); these were recorded as outcomes if the code was timestamped between the surgery date and hospital discharge. We utilized our previously developed and validated EHR-based computable phenotype algorithm to automatically determine the presence of AKI[1] as per Kidney Disease: Improving Global Outcomes (KDIGO)[2] standardized serum creatinine criteria. This AKI computable phenotype was specifically adapted to the PCORnet CDM, a methodology previously validated in a large multicenter cohort study within the OneFlorida network to characterize AKI epidemiology and trajectories.[3] The baseline serum creatinine was determined using preadmission measurements or the estimated serum creatinine.[1] An AKI event was confirmed if the creatinine measurement satisfying the KDIGO criteria occurred within the postoperative window (from surgery to discharge). In-hospital mortality was determined using the date of death, provided by institutional death records and a commercial death dataset from Datavant.[4]

Statistical Methods

We applied a powerful yet simple machine learning method, eXtreme Gradient Boosting (XGBoost), to develop models for predicting postoperative complications. We selected the parameters ‘n_estimators’, ‘gamma’, ‘max_depth’, ‘subsample’ and ‘colsample_bytree’ using 5-fold cross-validation on the development cohort. After determining the optimal parameters, we retrained the model using the entire development cohort.

We evaluated each model’smodel performance using area under the receiver operating characteristics curve (AUROC), area under the precision-recall curve (AUPRC), sensitivity, specificity, positive predictive value (PPV), negative predictive value (NPV), the Brier score and reliability plots. We employed bootstrap sampling and non-parametric methods to obtain 95% confidence interval (CI) for all performance metrics by thresholding the probability with the value optimized Youden index. We compared clinical characteristics and outcomes of patients across cohorts using the $\chi 2$ test for categorical variables and the Mann-Whitney U test for continuous variables. The threshold for statistical significance was set at less than 0.05 for 2-sided tests. P values for the family-wise error rate resulting from multiple comparisons were adjusted using the Bonferroni correction.

Error Subgroup Analysis

To systematically identify clinical “blind spots” — specific patient subgroups where predictive reliability is significantly reduced — we conducted a secondary error analysis using recursive partitioning. For each of the four outcomes (ICU admission, MV, AKI, and in-hospital mortality), a decision tree regressor was trained on the validation cohort.

To ensure the interpretability of the error profiles and focus on the most influential drivers of model instability, we restricted the input features to the top 10 most important features identified by the primary model,

© 2026 American Medical Association. All rights reserved, including those for text and data mining, AI training, and similar technologies.

rather than the full set of 99 variables. Within these inputs, the primary procedure and provider specialty features—which originally contained hundreds of unique levels—were mapped into 14 and 10 consolidated clinical categories, respectively. Specifically, primary procedure variable was categorized into transplant, cardiothoracic, vascular, neurological, colorectal, gastrointestinal (GI), oncology, orthopedic, urological, general surgery, gynecologic (GYN), ear nose throat (ENT), obstetrics (OB), and ophthalmology surgeries. Similarly, the provider specialty variable was consolidated into 10 representative categories: neuro/plastic, medical, general surgery, urology, orthopedics, anesthesia, obstetrics and gynecology (OB/GYN), ENT, ophthalmology, and support/other services. This grouping was performed to reduce feature dimensionality and ensure the resulting tree splits represent broad, clinically meaningful surgical pathways rather than granular, low-frequency individual procedures.

The decision tree was designed to predict the mean absolute error (MAE)—defined as the absolute difference between the model's calibrated predictions and the actual patient outcomes. By using the most impactful clinical features as splitting criteria, the tree automatically grouped patients into clusters with similar levels of model error. These final groupings create a transparent map that identifies exactly where the model is less reliable. In these high-error "blind spots," clinicians should use the model's risk scores with caution and rely more heavily on manual clinical judgment.

eFigures 4-7 present the error analysis results across the four outcomes. For postoperative ICU admission, the highest model instability (MAE=0.35) is concentrated among transferred patients undergoing neurological and transplant procedures (eFigure 4). This suggests a "blind spot" where the model may lack the granular, pre-transfer clinical history or the acute physiological markers required to predict ICU requirements in these highly complex cases. For posteropative MV, the highest model instability (MAE = 0.33) is identified in patients undergoing traditionally elective or lower-acuity surgical types (e.g., colorectal, ENT, GI, GYN, oncology, orthopedic) who require intensive preoperative monitoring (blood test count >9) (eFigure 5). This identifies a "hidden high-acuity" subgroup where clinical complexity deviates significantly from the typical baseline for these surgery types, leading to reduced model reliability. Significant error rates are also observed in transplant/vascular (MAE = 0.30) surgical pathways. For postoperative AKI, the highest model instability (MAE = 0.34) is concentrated in non-Hispanic patients undergoing high-acuity surgeries (e.g., cardiothoracic, colorectal, transplant, urological and vascular surgery) with elevated preoperative serum creatinine (≥1.2 mg/dL) (eFigure 6). This suggests that the model struggles to accurately predict AKI when baseline renal function is already impaired in high-risk surgical cohorts. For in-hospital mortality, the highest model instability (MAE = 0.19) is concentrated among transferred patients with a coagulopathy comorbidity (eFigure 7). This suggests that the model may not fully capture the physiological decline or the "failure to rescue" trajectory in patients arriving from outside facilities, particularly when coupled with comorbidities such as coagulopathy. Among all four outcomes, another comorbidity fluid electrolyte disorders (FED) served as a major warning sign for model error. Even in patient groups that the model usually predicts very accurately—such as those not transferred from other hospitals or those undergoing minor surgeries—the presence of FED caused a sudden drop in model reliability. This suggests that when a patient has a metabolic imbalance, their clinical path becomes much more unpredictable than the model's standard logic can handle.

In summary, while the models are highly accurate for the majority of patients, they exhibit consistent "blind spots" in three specific scenarios: 1). When patients are transferred from external facilities, the models often struggle because they lack the granular, pre-transfer history needed to assess the patient's full trajectory. 2). For patients in "low-risk" surgical specialties (like ENT or GYN), the models fail to recognize danger when it is masked by high-intensity monitoring or pre-existing metabolic issues. 3). In cases involving significant metabolic derangements (FED) or impaired baseline functions (such as high creatinine or coagulopathy), the models often underestimate the risk of sudden clinical decline or "failure to rescue."

© 2026 American Medical Association. All rights reserved, including those for text and data mining, AI training, and similar technologies.

eFigure 1. Flow Diagram for the identification of major surgery and the surgical cohort

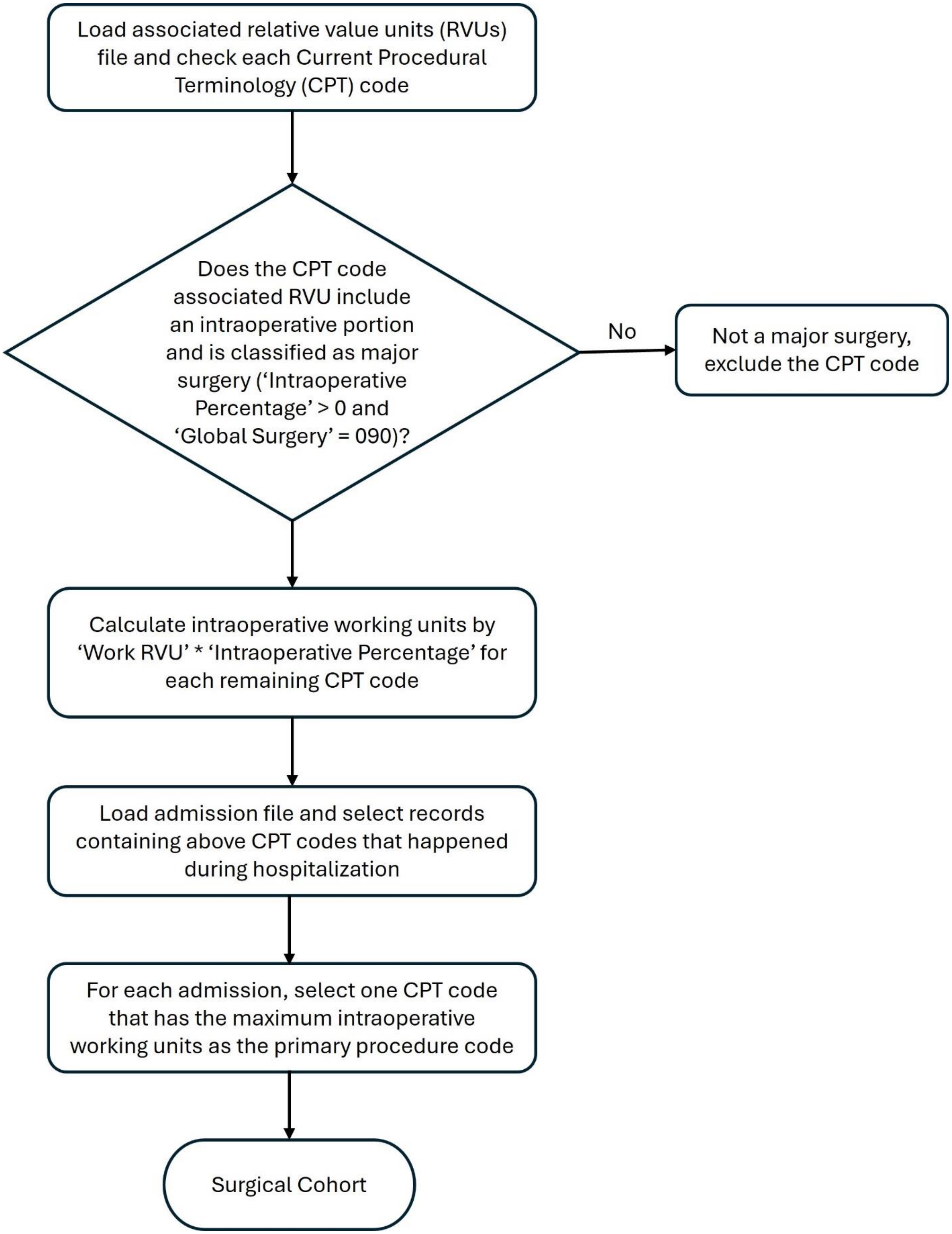

© 2026 American Medical Association. All rights reserved, including those for text and data mining, AI training, and similar technologies.

eFigure 2. Reliability plot demonstrating calibration performance on the calibration cohort

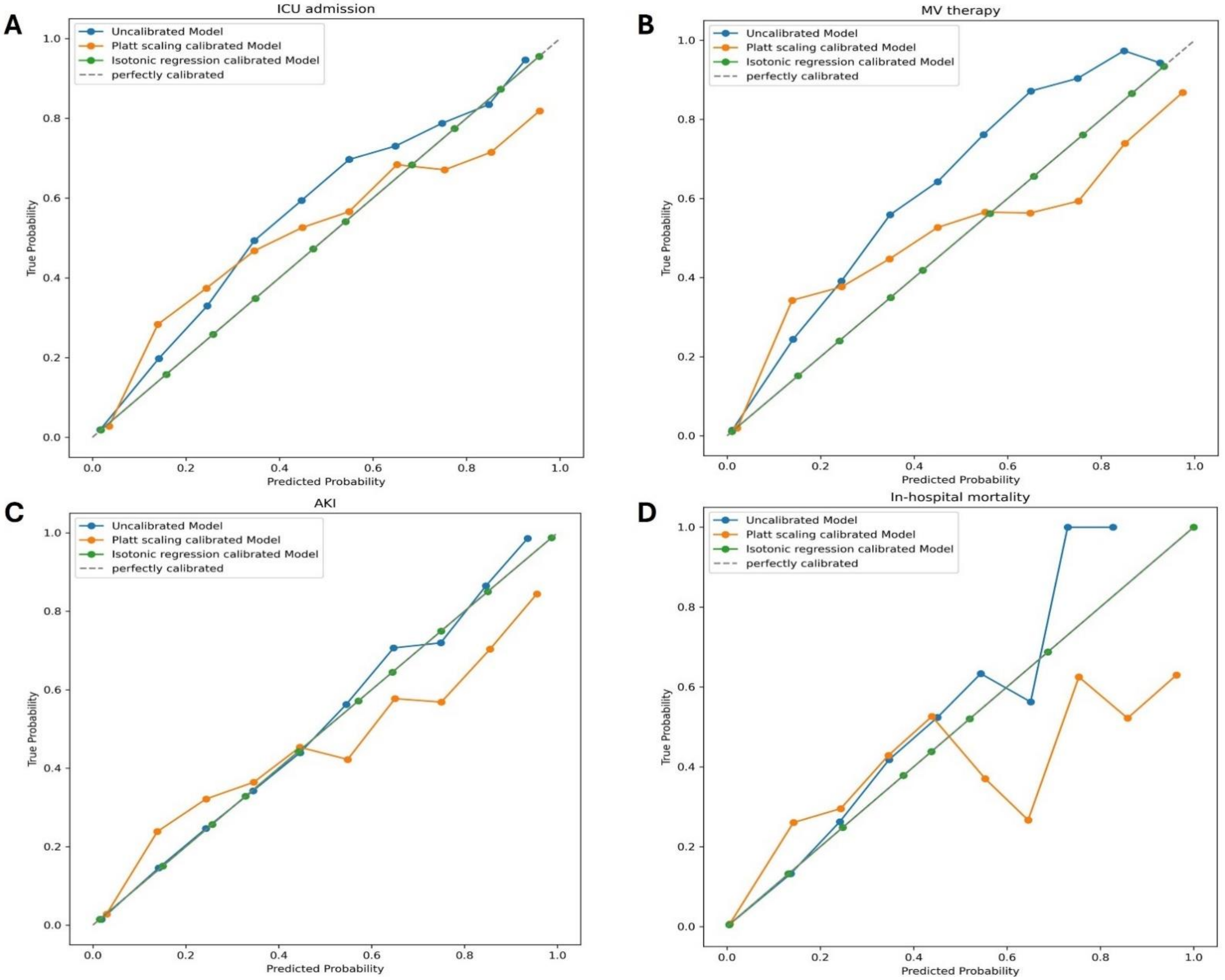

(A) Intensive care unit (ICU) admission; (B) Mechanical ventilation (MV) therapy; (C) Acute kidney injury (AKI); (D) In-hospital mortality. The diagonal line demonstrates the ideal calibration.

© 2026 American Medical Association. All rights reserved, including those for text and data mining, AI training, and similar technologies.

eFigure 3. Reliability plot demonstrating calibration performance on the validation cohort

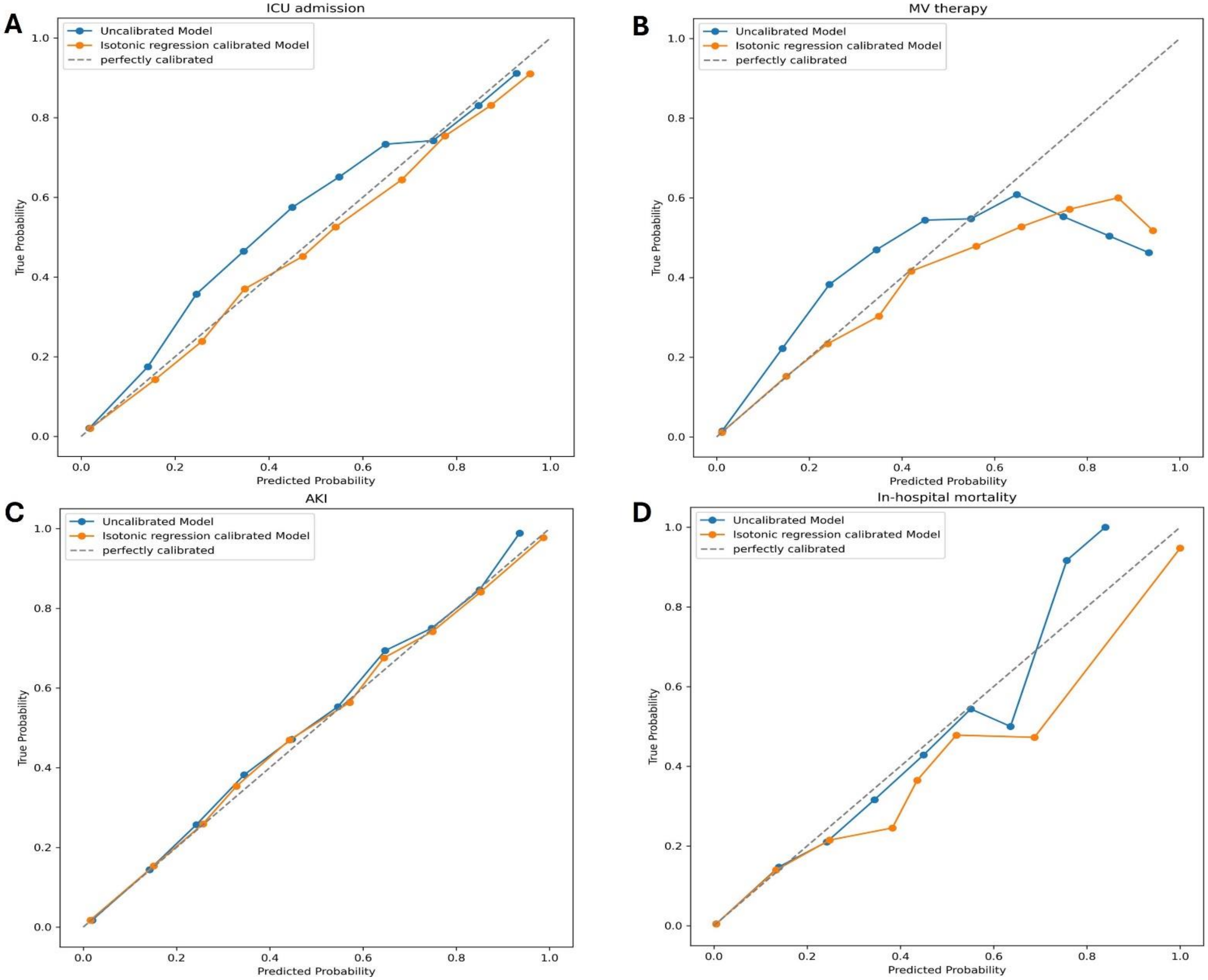

(A) Intensive care unit (ICU) admission; (B) Mechanical ventilation (MV) therapy; (C) Acute kidney injury (AKI); (D) In-hospital mortality. The diagonal line demonstrates the ideal calibration.

© 2026 American Medical Association. All rights reserved, including those for text and data mining, AI training, and similar technologies.

eFigure 4. Error subgroup analysis identifying clinical blind spots for intensive care unit (ICU) admission

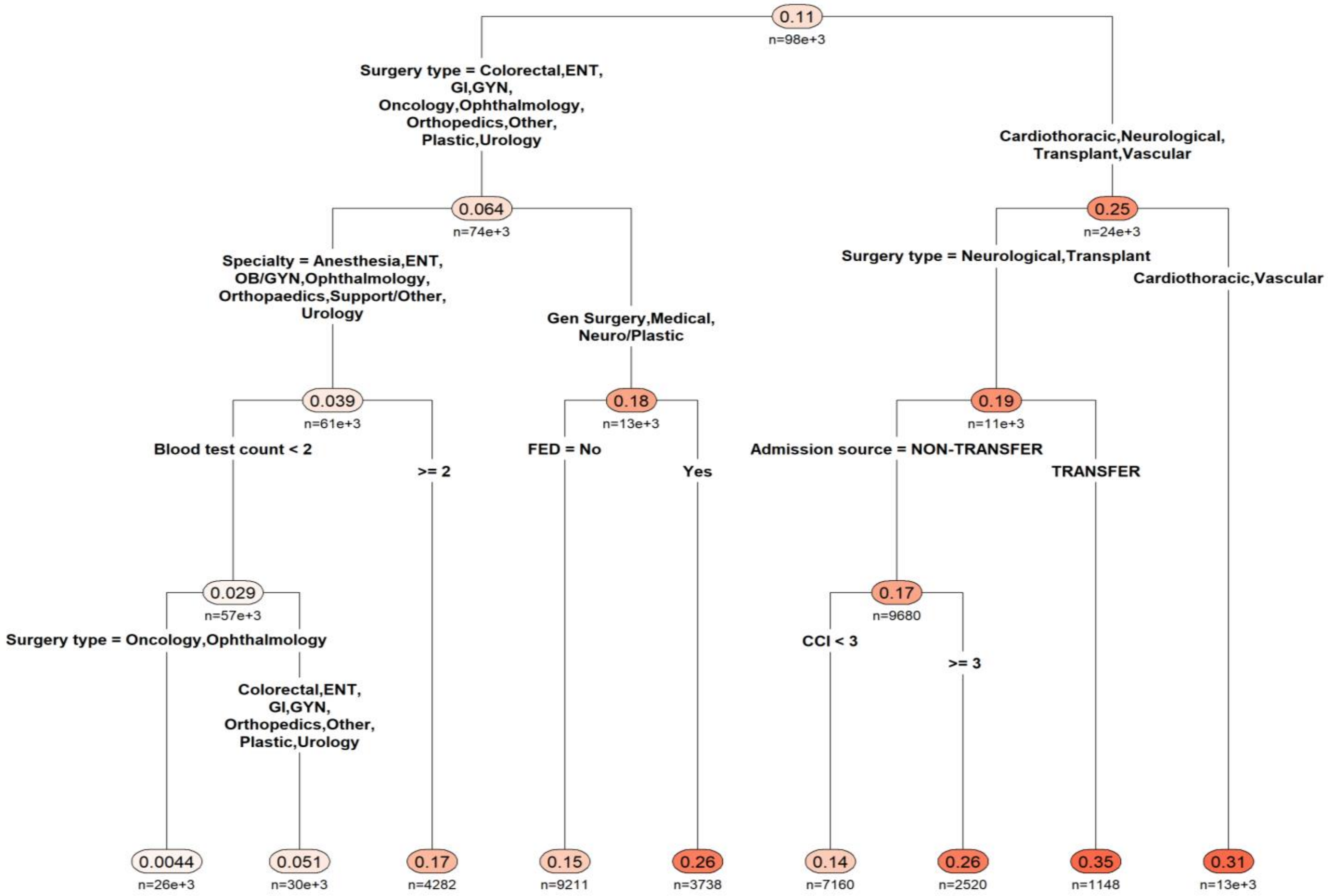

A decision tree regressor was utilized to partition the validation cohort based on the mean absolute error (MAE) of calibrated predictions. Each node displays the subgroup's mean MAE (top number) and total patient count (n, bottom number), with darker shading indicating higher error (reduced model reliability). The primary clinical blind spot (highest MAE=0.35) is concentrated among transferred patients undergoing neurological and transplant procedures. Conversely, the model exhibits its high reliability (low MAE=0.029) in patients with minimal preoperative testing (blood test count < 2) undergoing elective procedures such as oncology, ophthalmology, ENT (ear nose throat), GI (gastrointestinal), and GYN (gynecologic) surgeries.

© 2026 American Medical Association. All rights reserved, including those for text and data mining, AI training, and similar technologies.

eFigure 5. Error subgroup analysis identifying clinical blind spots for postoperative mechanical ventilation (MV)

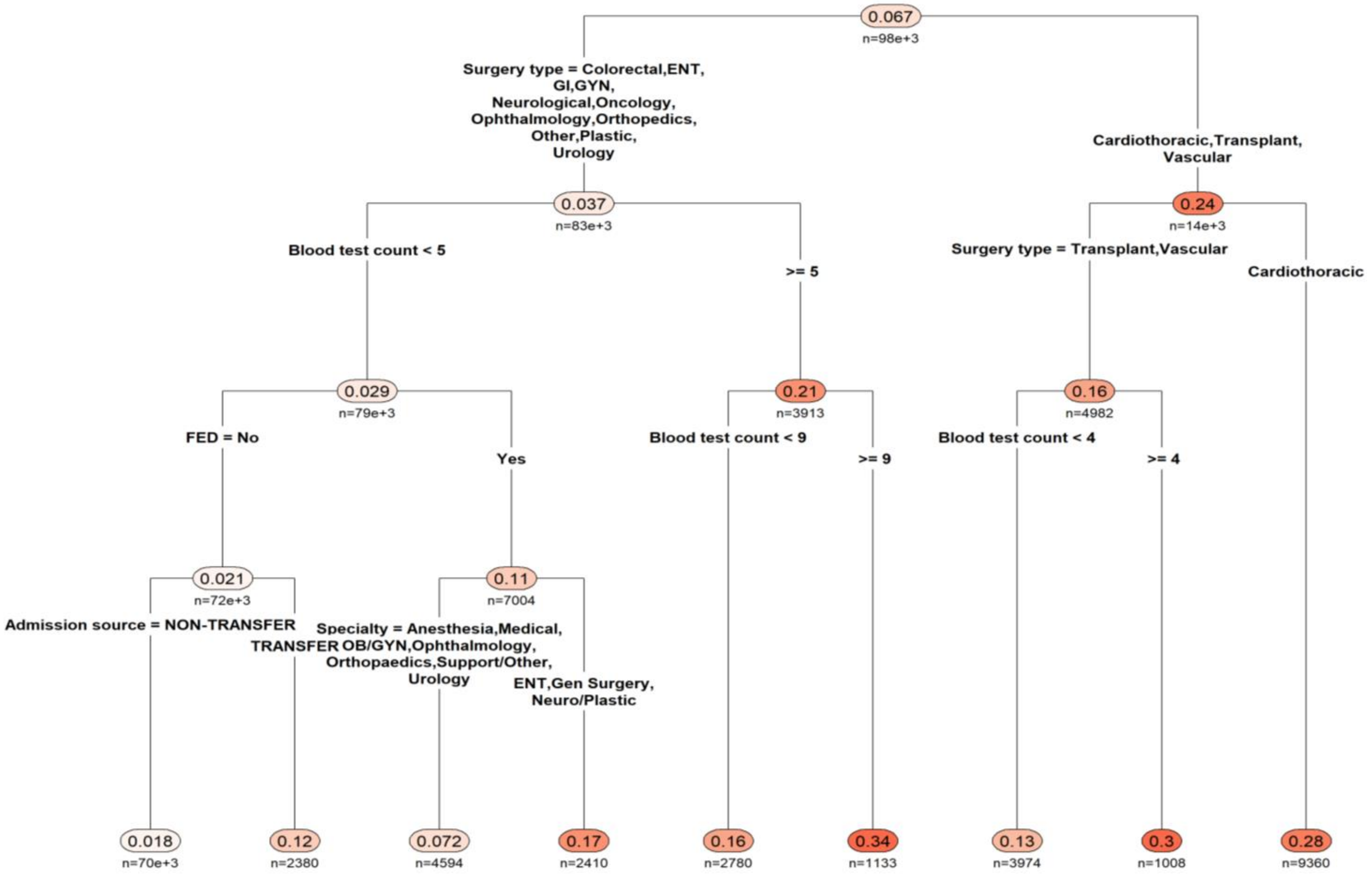

A decision tree regressor was utilized to partition the validation cohort based on the mean absolute error (MAE) of calibrated predictions. Each node displays the subgroup's mean MAE (top number) and total patient count (n, bottom number), with darker shading indicating higher error (reduced model reliability). The primary clinical blind spot (highest MAE=0.34) is concentrated in patients undergoing traditionally elective or low-acuity surgical types (e.g., oncology, ophthalmology, ENT [ear nose throat], GI [gastrointestinal], and GYN [gynecologic] surgeries), who requires intensive preoperative monitoring (blood test count > 9). Significant error rates are also observed in transplant/vascular (MAE = 0.30) surgical pathways. Conversely, the high-confidence zone (lowest MAE=0.018) is observed in non-transfer patients (n≈70,000) without fluid electrolyte disorders (FED) comorbidity and with minimal testing (blood test count < 5) undergoing elective surgeries.

© 2026 American Medical Association. All rights reserved, including those for text and data mining, AI training, and similar technologies.

eFigure 6. Error subgroup analysis identifying clinical blind spots for postoperative acute kidney injury (AKI)

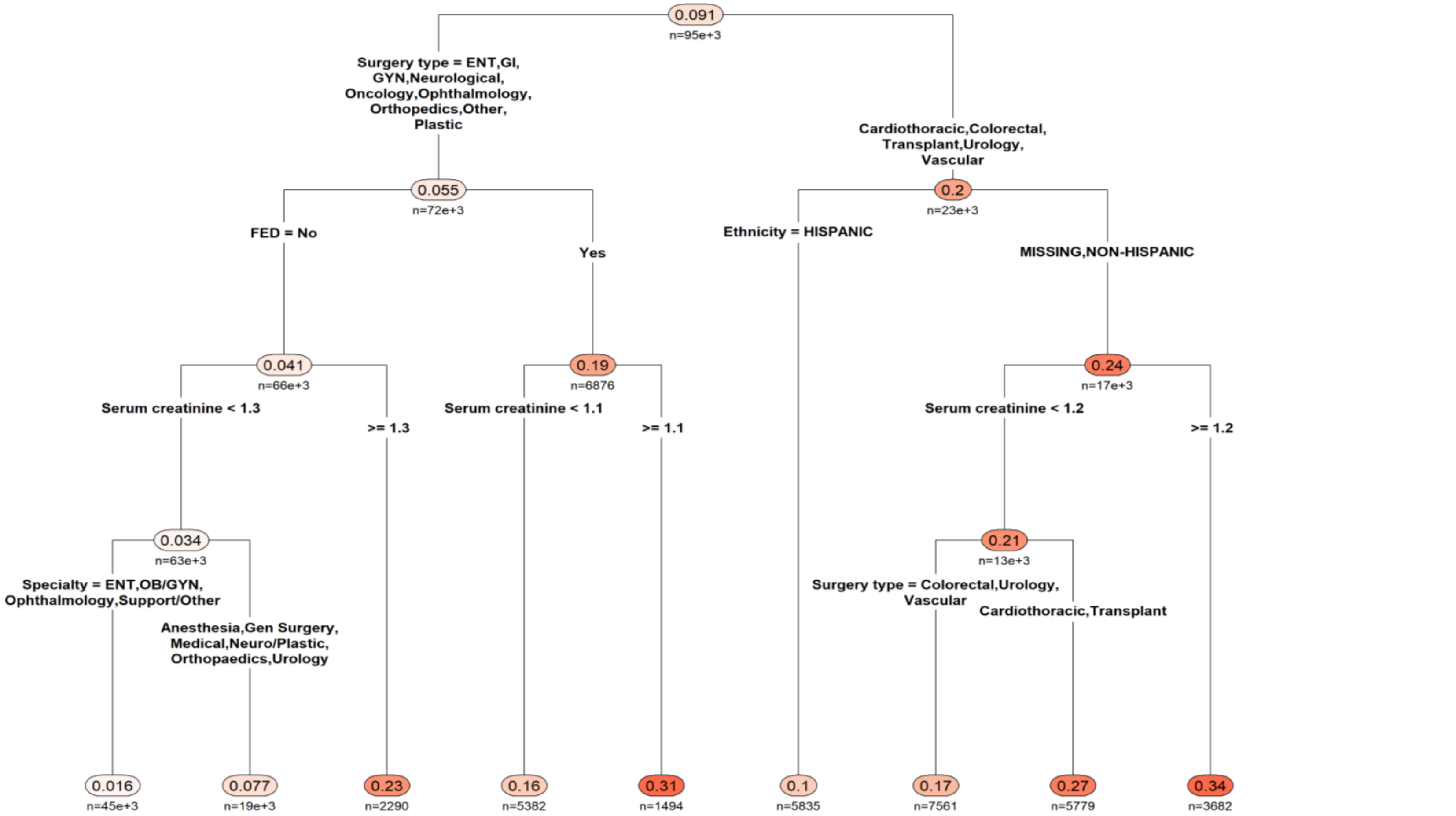

A decision tree regressor was utilized to partition the validation cohort based on the mean absolute error (MAE) of calibrated predictions. Each node displays the subgroup's mean MAE (top number) and total patient count (n, bottom number), with darker shading indicating higher error (reduced model reliability). The highest model instability (MAE = 0.34) is concentrated in non-Hispanic patients undergoing high-acuity surgeries (e.g., cardiothoracic, colorectal, transplant, urological and vascular surgery) with elevated preoperative serum creatinine (≥1.2 mg/dL). Conversely, the model is most reliable (lowest MAE = 0.016) for patients in low-intensity specialties and surgical types (e.g., ENT [ear nose throat], OB/GYN [Obstetrics and Gynecology], Ophthalmology) who have normal baseline renal function (serum creatinine < 1.3 mg/dL) and no fluid electrolyte disorders (FED).

© 2026 American Medical Association. All rights reserved, including those for text and data mining, AI training, and similar technologies.

eFigure 7. Error subgroup analysis identifying clinical blind spots for in-hospital mortality

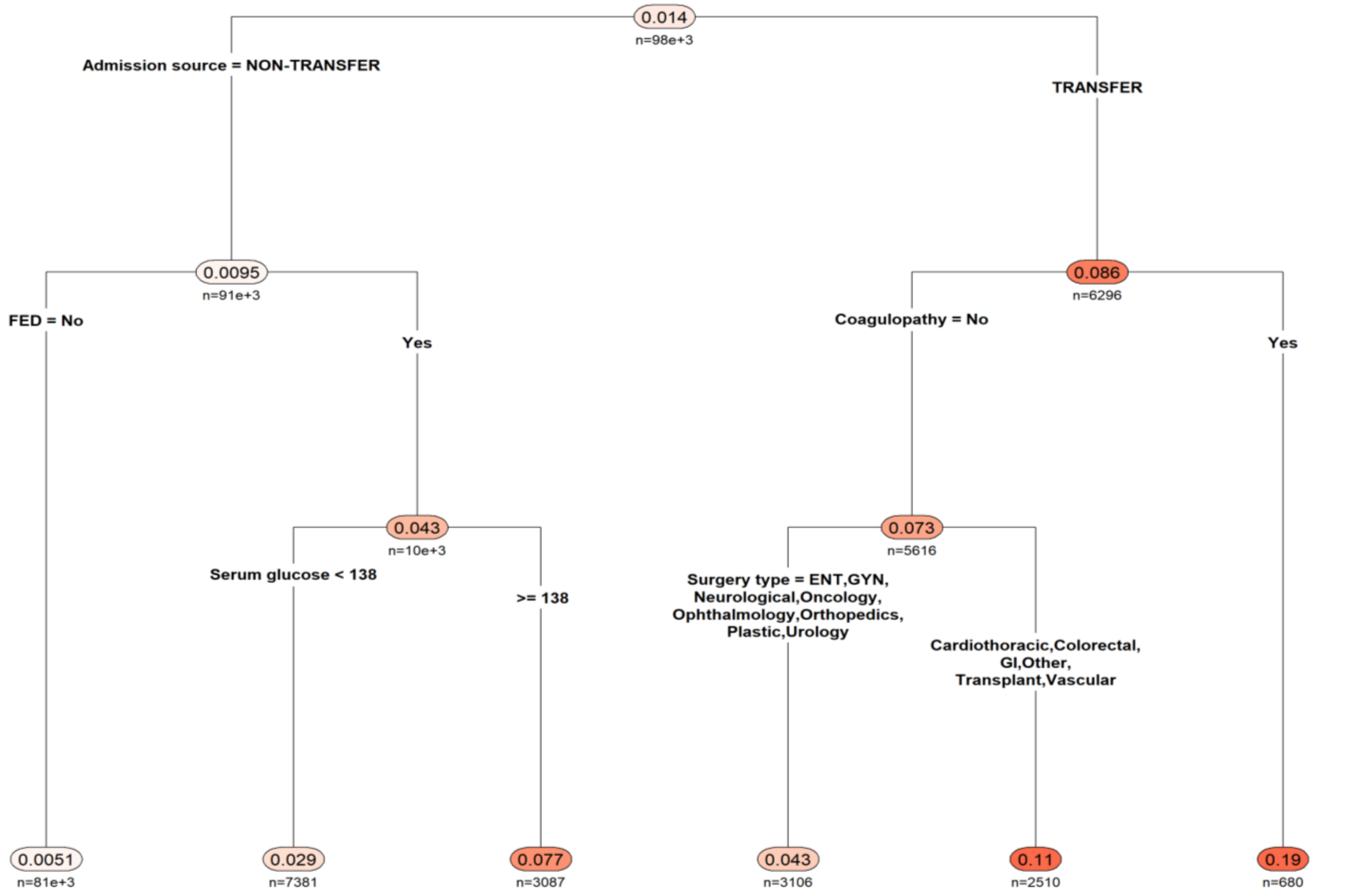

A decision tree regressor was utilized to partition the validation cohort based on the mean absolute error (MAE) of calibrated predictions. Each node displays the subgroup's mean MAE (top number) and total patient count (n, bottom number), with darker shading indicating higher error (reduced model reliability). The highest model instability (MAE = 0.19) is concentrated among transferred patients with a coagulopathy comorbidity. The model exhibits near-perfect reliability (lowest MAE=0.0051) in non-transfer patients (n≈81,000) without significant comorbidities (no fluid electrolyte disorders [FED]).

© 2026 American Medical Association. All rights reserved, including those for text and data mining, AI training, and similar technologies.

eTable 1. Administrative codes used for intensive care unit admission and mechanical ventilation

Code	Code type	Explanation
**Intensive care unit admission**		
99291	CPT	The first 30–74 minutes of critical care
99292	CPT	Each additional 30 minutes of critical care after the first 74 minutes
0188T	CPT	The first 30–74 minutes of remote, real-time, interactive, video-conferenced critical care.
0189T	CPT	Each additional 30 minutes of remote real-time interactive video-conferenced critical care.
99291	CPT	The first 30–74 minutes of critical care
**Mechanical ventilation**		
V46.1	ICD-9-CM Diagnosis	Dependence on respirator (ventilator)
V46.14	ICD-9-CM Diagnosis	Mechanical complication of respirator [ventilator]
Z99.11	ICD-10-CM Diagnosis	Dependence on respirator [ventilator] status
Z99.12	ICD-10-CM Diagnosis	Encounter for respirator [ventilator] dependence during power failure
J95.85	ICD-10-CM Diagnosis	Complication of respirator [ventilator]
J95.859	ICD-10-CM Diagnosis	Other complication of respirator [ventilator]
96.7	ICD-9-CM Procedure	Other continuous invasive mechanical ventilation
96.70	ICD-9-CM Procedure	Continuous invasive mechanical ventilation of an unspecified duration
96.71	ICD-9-CM Procedure	Continuous invasive mechanical ventilation for less than 96 consecutive hours
96.72	ICD-9-CM Procedure	Continuous invasive mechanical ventilation for 96 consecutive hours or more
5A1935Z	ICD-10-PCS Procedure	Respiratory ventilation, less than 24 consecutive hours
5A1945Z	ICD-10-PCS Procedure	Respiratory ventilation, 24-96 consecutive hours
5A1955Z	ICD-10-PCS Procedure	Respiratory ventilation for a duration of greater than 96 consecutive hours
94657	CPT	Ventilation assist and management, initiation of pressure or volume preset ventilators for assisted or controlled breathing; nursing facility
94656	CPT	Ventilation assist and management, initiation of pressure or volume preset ventilators for assisted or controlled breathing; hospital
94003	CPT	The ongoing daily management of the ventilator for a patient in a hospital inpatient or observation setting. It is used for each subsequent day after the initial day of ventilation
94002	CPT	The initiation and management of pressure- or volume-preset ventilators for assisted or controlled breathing
94004	CPT	The daily management of a ventilator for a patient in a nursing facility

© 2026 American Medical Association. All rights reserved, including those for text and data mining, AI training, and similar technologies.

eTable 2. Input features used in models

Feature	Model Published	Model in This Study	Type	Missingness (%)
**Demographics**				
Age	X	X	Numerical	0
Sex	X	X	Binary	0
Race	X	X	Categorical	3
Ethnicity	X	X	Categorical	3
Native Language Spoken	X	X	Binary	0
Marital Status	X		Categorical	NA
Smoking Status	X		Categorical	82
Body Mass Index	X	X	Numerical	1
**Neighborhood and Socioeconomic**				
ZIP Code of home address	X		Categorical	56
County of home address	X		Categorical	56
Rural or city at patient residential area	X		Binary	56
Distance of residence to hospital, km	X		Numerical	56
median total income at patient residential area, USD	X		Numerical	56
prevalence of African American residents living below poverty at patient residential area, %	X		Numerical	56
prevalence of Hispanic residents living below poverty at patient residential area, %	X		Numerical	56
Prevalence of residents living below poverty at patient residential area, %	X		Numerical	56
Insurance paying the bills	X	X	Categorical	0
**Comorbidities**				
Charlson comorbidity index	X	X	Categorical	0
Myocardial Infarction	X	X	Binary	0
Congestive Heart Failure	X	X	Binary	0
Cerebrovascular Disease	X	X	Binary	0
Chronic Pulmonary Disease	X	X	Binary	0
Peripheral Vascular Disease	X	X	Binary	0
Cancer	X	X	Binary	0
Liver Disease	X	X	Binary	0
Valvular Disease	X	X	Binary	0
Coagulopathy	X	X	Binary	0
Weight Loss	X	X	Binary	0
alcohol abuse or drug	X	X	Binary	0
Fluid and electrolyte disorders	X	X	Binary	0
Chronic anemia	X	X	Binary	0
Hypertension	X	X	Binary	0
Obesity	X	X	Binary	0

© 2026 American Medical Association. All rights reserved, including those for text and data mining, AI training, and similar technologies.

Feature	Model Published	Model in This Study	Type	Missingness (%)
Diabetes	X	X	Binary	0
Metastatic Carcinoma	X	X	Binary	0
Depression	X	X	Binary	0
CKD status at admission	X	X	Categorical	0
Reference estimated glomerular filtration rate	X	X	Numerical	37
**Admission Characteristics**				
Admission Source	X	X	Binary	0
Admission Day	X	X	Categorical	0
Admission Month	X	X	Categorical	0
emergent or elective admission	X		Binary	NA
Yes, if the admission we happened at night	X	X	Binary	0
Medicine or surgery admitting	X		Categorical	NA
**Operative Variables**				
Current Procedural Terminology code of the primary procedure	X	X	Categorical	0
Scheduled post operation location	X		Binary	NA
Scheduled room is trauma room or not	X		Binary	NA
Scheduled anesthesia Type	X		Binary	NA
Scheduled surgery room	X		Categorical	NA
Surgery Type	X		Categorical	NA
ID of Attending Surgeon	X		Categorical	0
Specialty		X	Categorical	15
Time from Admission to Surgery, days	X		Numerical	NA
**Medication History**				
Indicator of receiving Betablockers within one year before admission date	X	X	Binary	0
Indicator of receiving Diuretics within one year before admission date	X	X	Binary	0
Indicator of receiving statin within one year before admission date	X	X	Binary	0
Indicator of receiving Aspirin within one year before admission date	X	X	Binary	0
Indicator of receiving ACE Inhibitors within one year before admission date	X	X	Binary	0
Indicator of receiving vasopressors or inotropes within one year before admission date	X	X	Binary	0
Indicator of receiving Bicarbonate within one year before admission date	X	X	Binary	0
Indicator of receiving Antiemetic within one year before admission date	X	X	Binary	0
Indicator of receiving Aminoglycosides within one year before admission date	X	X	Binary	0

© 2026 American Medical Association. All rights reserved, including those for text and data mining, AI training, and similar technologies.

Feature	Model Published	Model in This Study	Type	Missingness (%)
Indicator of receiving Vancomycin within one year before admission date	X	X	Binary	0
No of nephrotoxic drugs received within one year before admission date	X	X	Binary	0
**Historical Laboratory Results**				
Automated urinalysis, urine protein presence within 365 days prior to surgery, mg/dL	X		Categorical	100
Automated urinalysis, urine hemoglobin within 8-365 days prior to surgery, mg/dL	X		Categorical	100
Min hemoglobin within 8-365 days prior to surgery, g/dl	X	X	Numerical	58
Max hemoglobin within 8-365 days prior to surgery, g/dl	X	X	Numerical	58
Average of hemoglobin within 8-365 days prior to surgery, g/dl	X	X	Numerical	58
Number of hemoglobin tests within 8-365 days prior to surgery	X	X	Numerical	0
Number of urine hemoglobin tests within 8-365 days prior to surgery	X		Categorical	0
**Preoperative Laboratory Results**				
Automated urinalysis, urine glucose within 7 days prior to surgery, mg/dL	X		Categorical	100
Automated urinalysis, urine hemoglobin within 7 days prior to surgery, mg/dL	X		Categorical	100
Min hemoglobin within 7 days prior to surgery, g/dl	X	X	Numerical	59
Max hemoglobin within 7 days prior to surgery, g/dl	X	X	Numerical	59
Average of hemoglobin within 7 days prior to surgery, g/dl	X	X	Numerical	59
Variance of hemoglobin within 7 days prior to surgery, g/dl	X	X	Numerical	59
Number of hemoglobin tests within 7 days prior to surgery	X	X	Numerical	0
Min of Serum Calcium, mmol/L	X	X	Numerical	61
Max of Serum Calcium, mmol/L	X	X	Numerical	61
Average of Serum Calcium, mmol/L	X	X	Numerical	61
Variance of Serum Calcium, mmol/L	X	X	Numerical	61
Count of Serum Calcium in blood test	X		Numerical	0
Average of anion gap in blood, mmol/L	X	X	Numerical	64
Count of anion gap in blood test	X		Numerical	0
Min of White Blood Cell in blood, thou/uL	X	X	Numerical	59
Max of White Blood Cell in blood, thou/uL	X	X	Numerical	59

© 2026 American Medical Association. All rights reserved, including those for text and data mining, AI training, and similar technologies.

Feature	Model Published	Model in This Study	Type	Missingness (%)
Average of White Blood Cell in blood, thou/uL	X	X	Numerical	59
Variance of White Blood Cell in blood, thou/uL	X	X	Numerical	59
Count of White Blood Cell in blood test	X		Numerical	0
Min of Hematocrit in blood, %	X	X	Numerical	59
Average of Hematocrit in blood, %	X	X	Numerical	59
Variance of Hematocrit in blood, %	X	X	Numerical	59
Count of Hematocrit in blood test	X		Numerical	0
Max of Serum Red Blood Cell, Million/uL	X	X	Numerical	59
Average of Serum Red Blood Cell, Million/uL	X	X	Numerical	59
Max of the amount of hemoglobin relative to the size of the cell in blood, g/dL	X	X	Numerical	59
Average of the amount of hemoglobin relative to the size of the cell in blood, g/dL	X	X	Numerical	59
Min of Glucose in blood, mg/dL	X	X	Numerical	60
Max of Glucose in blood, mg/dL	X	X	Numerical	60
Average of Glucose in blood, mg/dL	X	X	Numerical	60
Count of Glucose in blood test	X	X	Numerical	0
Min of Serum CO2 , mmol/L	X	X	Numerical	72
Max of Serum CO2, mmol/L	X	X	Numerical	72
Average of Serum CO2, mmol/L	X	X	Numerical	72
Variance of Serum CO2, mmol/L	X	X	Numerical	72
Count of Serum CO2 test	X		Numerical	0
Min of Urea nitrogen in blood, mg/dL	X	X	Numerical	60
Max of Urea nitrogen in blood, mg/dL	X	X	Numerical	60
Average of Urea nitrogen in blood, mg/dL	X	X	Numerical	60
Variance of Urea nitrogen in blood, mg/dL	X	X	Numerical	60
Count of Urea nitrogen in blood test	X		Numerical	0
Min of Urea Nitrogen-Creatinine ratio	X		Numerical	86
Max of Urea Nitrogen-Creatinine ratio	X		Numerical	86
Average of Urea Nitrogen-Creatinine ratio	X		Numerical	86
Variance of Urea Nitrogen-Creatinine ratio	X		Numerical	86
Count of Urea Nitrogen-Creatinine ratio	X		Numerical	0
Max of Serum Sodium, mmol/L	X	X	Numerical	60
Average of Serum Sodium, mmol/L	X	X	Numerical	60
Count of Serum Sodium test	X		Numerical	0

© 2026 American Medical Association. All rights reserved, including those for text and data mining, AI training, and similar technologies.

Feature	Model Published	Model in This Study	Type	Missingness (%)
Average of Potassium in serum, mmol/L	X	X	Numerical	60
Count of Potassium in serum test	X		Numerical	0
Max of Red cell distribution width in Blood, %	X	X	Numerical	59
Average of Red cell distribution width in Blood, %	X	X	Numerical	59
Variance of Red cell distribution width in Blood, %	X	X	Numerical	59
Min of platelet in blood, thou/uL	X	X	Numerical	59
Max of platelet in blood, thou/uL	X	X	Numerical	59
Average of platelet in blood, thou/uL	X	X	Numerical	59
Variance of platelet in blood, thou/uL	X	X	Numerical	59
Min of Serum creatinine, mg/dL	X	X	Numerical	60
Max of Serum creatinine, mg/dL	X	X	Numerical	60
Average of Serum creatinine, mg/dL	X	X	Numerical	60
Variance of Serum creatinine, mg/dL	X	X	Numerical	60
Count of Serum creatinine test	X		Numerical	60
Max of chloride in Serum, mmol/L	X	X	Numerical	60
Average of chloride in Serum, mmol/L	X	X	Numerical	60
Variance of chloride in Serum, mmol/L	X	X	Numerical	60
Count of chloride in Serum test	X		Numerical	0

© 2026 American Medical Association. All rights reserved, including those for text and data mining, AI training, and similar technologies.

eTable 3. Brier score assessment of calibration methods in the calibration cohort

Outcome	Method	Score[a] before calibration	Score after calibration
Intensive care unit admission	Platt scaling	0.0535	0.0549
	Isotonic regression	0.0535	0.0515
Mechanical ventilation	Platt scaling	0.0319	0.0319
	Isotonic regression	0.0319	0.0294
Acute kidney injury	Platt scaling	0.0431	0.0449
	Isotonic regression	0.0431	0.0428
Hospital mortality	Platt scaling	0.00744	0.00789
	Isotonic regression	0.00744	0.00735

[a] The Brier score ranges from 0 to 1. Lower Brier score indicates better model performance, meaning the predicted probabilities are closer to the actual outcomes.

© 2026 American Medical Association. All rights reserved, including those for text and data mining, AI training, and similar technologies.

eTable 4. Model performance measurements for postoperative complications with 95% confidence interval in the validation cohort stratified by sex

Complications	Sex	AUROC	AUPRC	Sensitivity	Specificity	PPV	NPV	Brier score
ICU admission	Female	0.93 (0.93-0.94)	0.58 (0.57-0.6)	0.86 (0.84-0.87)	0.85 (0.84-0.86)	0.31 (0.31-0.34)	0.99 (0.98-0.99)	0.04 (0.04-0.05)
	Male	0.92 (0.92-0.92)	0.63 (0.62-0.65)	0.87 (0.84-0.90)	0.81 (0.79-0.84)	0.39 (0.36-0.43)	0.98 (0.97-0.98)	0.07 (0.06-0.07)
MV	Female	0.94 (0.94-0.95)	0.41 (0.39-0.43)	0.86 (0.84-0.91)	0.89 (0.84-0.91)	0.23 (0.18-0.26)	0.99 (0.99-1.0)	0.03 (0.03-0.03)
	Male	0.92 (0.92-0.93)	0.45 (0.43-0.47)	0.87 (0.85-0.89)	0.84 (0.82-0.87)	0.27 (0.25-0.30)	0.99 (0.99-0.99)	0.05 (0.04-0.05)
AKI	Female	0.93 (0.92-0.93)	0.54 (0.52-0.56)	0.86 (0.84-0.92)	0.83 (0.78-0.84)	0.25 (0.21-0.27)	0.99 (0.99-0.99)	0.04 (0.04-0.04)
	Male	0.90 (0.90-0.91)	0.54 (0.52-0.55)	0.86 (0.81-0.88)	0.77 (0.76-0.82)	0.26 (0.25-0.29)	0.98 (0.98-0.99)	0.05 (0.05-0.06)
In-hospital mortality	Female	0.96 (0.95-0.97)	0.24 (0.20-0.29)	0.92 (0.87-0.98)	0.87 (0.80-0.92)	0.04 (0.03-0.07)	1.0 (1.0-1.0)	0.01 (0.00-0.01)
	Male	0.94 (0.93-0.95)	0.26 (0.22-0.30)	0.88 (0.82-0.92)	0.84 (0.81-0.89)	0.05 (0.04-0.08)	1.0 (1.0-1.0)	0.01 (0.01-0.01)

Abbreviations: AUROC, area under the receiver operating characteristic curve; AUPRC, area under the precision-recall curve; PPV, positive predictive value; NPV, negative predictive value; ICU, intensive care unit; MV, mechanical ventilation; AKI, acute kidney injury.

© 2026 American Medical Association. All rights reserved, including those for text and data mining, AI training, and similar technologies.

eTable 5. Model performance measurements for postoperative complications with 95% confidence interval in the validation cohort stratified by race

Complications	Race	AUROC	AUPRC	Sensitivity	Specificity	PPV	NPV	Brier score
ICU admission	African American	0.91 (0.90-0.91)	0.61 (0.58-0.63)	0.83 (0.80-0.86)	0.81 (0.79-0.83)	0.34 (0.32-0.37)	0.98 (0.97-0.98)	0.06 (0.06-0.06)
	Non-African American	0.93 (0.93-0.93)	0.62 (0.60-0.63)	0.88 (0.85-0.90)	0.83 (0.81-0.86)	0.36 (0.34-0.39)	0.98 (0.98-0.99)	0.05 (0.05-0.05)
MV	African American	0.91 (0.90-0.92)	0.47 (0.44-0.51)	0.81 (0.78-0.85)	0.87 (0.84-0.88)	0.27 (0.23-0.28)	0.99 (0.99-0.99)	0.04 (0.04-0.04)
	Non-African American	0.94 (0.94-0.94)	0.43 (0.42-0.45)	0.89 (0.87-0.90)	0.86 (0.86-0.88)	0.25 (0.24-0.27)	0.99 (0.99-0.99)	0.04 (0.03-0.04)
AKI	African American	0.90 (0.89-0.90)	0.56 (0.53-0.58)	0.83 (0.81-0.89)	0.79 (0.73-0.81)	0.30 (0.26-0.32)	0.98 (0.97-0.98)	0.06 (0.06-0.06)
	Non-African American	0.92 (0.92-0.92)	0.53 (0.52-0.54)	0.86 (0.84-0.89)	0.81 (0.79-0.83)	0.25 (0.23-0.26)	0.99 (0.99-0.99)	0.04 (0.04-0.04)
In-hospital mortality	African American	0.94 (0.92-0.95)	0.34 (0.26-0.42)	0.90 (0.82-0.95)	0.84 (0.81-0.89)	0.05 (0.04-0.08)	1.0 (1.0-1.0)	0.01 (0.01-0.01)
	Non-African American	0.95 (0.95-0.96)	0.23 (0.20-0.26)	0.91 (0.84-0.93)	0.85 (0.84-0.91)	0.04 (0.04-0.07)	1.0 (1.0-1.0)	0.01 (0.01-0.01)

Abbreviations: AUROC, area under the receiver operating characteristic curve; AUPRC, area under the precision-recall curve; PPV, positive predictive value; NPV, negative predictive value; ICU, intensive care unit; MV, mechanical ventilation; AKI, acute kidney injury.

© 2026 American Medical Association. All rights reserved, including those for text and data mining, AI training, and similar technologies.

eTable 6. Model performance measurements for postoperative complications with 95% confidence interval in the validation cohort stratified by age

Complications	Age	AUROC	AUPRC	Sensitivity	Specificity	PPV	NPV	Brier score
ICU admission	≤65	0.93 (0.92-0.93)	0.62 (0.61-0.64)	0.86 (0.85-0.87)	0.84 (0.83-0.86)	0.37 (0.35-0.39)	0.98 (0.98-0.98)	0.05 (0.05-0.06)
	>65	0.93 (0.92-0.93)	0.60 (0.58-0.62)	0.91 (0.84-0.91)	0.79 (0.78-0.85)	0.32 (0.31-0.39)	0.99 (0.98-0.99)	0.06 (0.05-0.06)
MV	≤65	0.94 (0.93-0.94)	0.46 (0.44-0.48)	0.87 (0.85-0.88)	0.87 (0.87-0.88)	0.26 (0.25-0.28)	0.99 (0.99-0.99)	0.04 (0.03-0.04)
	>65	0.94 (0.93-0.94)	0.40 (0.38-0.43)	0.87 (0.86-0.90)	0.87 (0.84-0.87)	0.25 (0.22-0.26)	0.99 (0.99-0.99)	0.04 (0.04-0.04)
AKI	≤65	0.92 (0.91-0.92)	0.52 (0.51-0.54)	0.84 (0.83-0.89)	0.84 (0.78-0.84)	0.25 (0.20-0.26)	0.99 (0.99-0.99)	0.04 (0.04-0.04)
	>65	0.91 (0.90-0.91)	0.55 (0.53-0.56)	0.85 (0.83-0.89)	0.79 (0.76-0.81)	0.28 (0.26-0.30)	0.98 (0.98-0.99)	0.06 (0.05-0.06)
In-hospital mortality	≤65	0.96 (0.95-0.96)	0.29 (0.25-0.34)	0.92 (0.88-0.94)	0.87 (0.87-0.89)	0.05 (0.04-0.06)	1.0 (1.0-1.0)	0.01 (0.01-0.01)
	>65	0.93 (0.93-0.94)	0.22 (0.18-0.26)	0.84 (0.81-0.91)	0.88 (0.79-0.88)	0.07 (0.04-0.07)	1.0 (1.0-1.0)	0.01 (0.01-0.01)

Abbreviations: AUROC, area under the receiver operating characteristic curve; AUPRC, area under the precision-recall curve; PPV, positive predictive value; NPV, negative predictive value; ICU, intensive care unit; MV, mechanical ventilation; AKI, acute kidney injury.

© 2026 American Medical Association. All rights reserved, including those for text and data mining, AI training, and similar technologies.

eTable 7. Sensitivity analysis adding personalized feature surgeon ID: model performance measurements for postoperative complications with 95% confidence interval in the validation cohort

Complications	With surgeon ID	AUROC	AUPRC	Sensitivity	Specificity	PPV	NPV	Brier score
ICU admission	N	0.93 (0.93-0.93)	0.61 (0.60-0.63)	0.87 (0.85-0.89)	0.82 (0.81-0.84)	0.35 (0.34-0.37)	0.98 (0.98-0.99)	0.06 (0.05-0.06)
	Y	0.93 (0.93-0.93)	0.62 (0.61-0.63)	0.87 (0.84-0.88)	0.84 (0.82-0.86)	0.37 (0.35-0.40)	0.98 (0.98-0.98)	0.05 (0.05-0.05)
MV	N	0.94 (0.93-0.94)	0.44 (0.42-0.45)	0.88 (0.86-0.89)	0.86 (0.86-0.88)	0.25 (0.24-0.27)	0.99 (0.99-0.99)	0.04 (0.04-0.04)
	Y	0.94 (0.93-0.94)	0.42 (0.41-0.44)	0.88 (0.87-0.90)	0.86 (0.83-0.86)	0.24 (0.22-0.25)	0.99 (0.99-0.99)	0.04 (0.04-0.04)
AKI	N	0.92 (0.91-0.92)	0.54 (0.52-0.55)	0.86 (0.85-0.88)	0.8 (0.78-0.82)	0.25 (0.24-0.27)	0.99 (0.99-0.99)	0.05 (0.05-0.05)
	Y	0.92 (0.92-0.92)	0.54 (0.53-0.56)	0.89 (0.84-0.90)	0.78 (0.78-0.83)	0.24 (0.24-0.28)	0.99 (0.99-0.99)	0.05 (0.04-0.05)
In-hospital mortality	N	0.95 (0.94-0.95)	0.25 (0.22-0.29)	0.91 (0.85-0.93)	0.84 (0.84-0.91)	0.04 (0.04-0.07)	1.0 (1.0-1.0)	0.01 (0.01-0.01)
	Y	0.95 (0.94-0.95)	0.25 (0.21-0.28)	0.87 (0.86-0.93)	0.87 (0.83-0.88)	0.05 (0.04-0.06)	1.0 (1.0-1.0)	0.01 (0.01-0.01)

Abbreviations: AUROC, area under the receiver operating characteristic curve; AUPRC, area under the precision-recall curve; PPV, positive predictive value; NPV, negative predictive value; ICU, intensive care unit; MV, mechanical ventilation; AKI, acute kidney injury.

© 2026 American Medical Association. All rights reserved, including those for text and data mining, AI training, and similar technologies.

Reference

1. Ozrazgat-Baslanti T, Ren Y, Adiyeke E, et al. Development and validation of a race-agnostic computable phenotype for kidney health in adult hospitalized patients. *PLoS One*. 2024;19(4):e0299332. doi:10.1371/journal.pone.0299332
2. Group K. KDIGO clinical practice guideline for acute kidney injury. *Kidney Int Suppl*. 2012;2:1.
3. Adiyeke E, Ren Y, Fogel S, et al. Epidemiology, trajectories and outcomes of acute kidney injury among hospitalized patients: a large retrospective multicenter cohort study. *J Nephrol*. Jul 2025;38(6):1673-1682. doi:10.1007/s40620-025-02234-4
4. Hogan WR, Shenkman EA, Robinson T, et al. The OneFlorida Data Trust: a centralized, translational research data infrastructure of statewide scope. *J Am Med Inform Assoc*. Mar 15 2022;29(4):686-693. doi:10.1093/jamia/ocab221

© 2026 American Medical Association. All rights reserved, including those for text and data mining, AI training, and similar technologies.
