# Supplementary material for: MySurgeryRisk Model Predictions of Postoperative Complications and Mortality
Source: JAMA Surg. 2026 Apr 29;161(6):619–27. doi: 10.1001/jamasurg.2026.1112 (PMC13130070; doi:10.1001/jamasurg.2026.1112)
Supplement: Supplement 2. — Data sharing statement [file jamasurg-e261112-s002.pdf]

Data Sharing Statement

Ren. MySurgeryRisk Model Predictions of Postoperative Complications and Mortality. *JAMA Surg*. Published April 29, 2026. doi:10.1001/jamasurg.2026.1112

Data
Data available: No

Additional Information
Explanation for why data not available: Data availability statement The data used in this study were obtained from the OneFlorida+ Clinical Research Network (https://onefloridaconsortium.org/). Researchers may access the data with an approved study protocol and data use agreement (DUA) at https://onefloridaconsortium.org/front-door/prep-to-research-data-query/.
